# Pregnancy Is Not Associated with an Increased Risk of Decompensation, Transplant, or Death in Compensated Cirrhosis

**DOI:** 10.1155/2022/9985226

**Published:** 2022-07-06

**Authors:** Monica Mullin, Maya Djerboua, Monika Sarkar, Jacquie Lu, Maria P. Velez, Susan Brogly, Norah A. Terrault, Jennifer A. Flemming

**Affiliations:** ^1^Department of Medicine, Queen's University, Canada; ^2^ICES, Queen's University, Canada; ^3^Department of Medicine, University of California San Francisco, USA; ^4^Departments of Obstetrics and Gynecology, Queen's University, Canada; ^5^Department of Public Health Sciences, Queen's University, Canada; ^6^Department of Surgery, Queen's University, Canada; ^7^Department of Medicine, University of Southern California, USA

## Abstract

**Background and Aims:**

Childbirth in women with cirrhosis is increasing and associated with a higher risk of perinatal outcomes compared to the general population. Whether pregnancy influences the risk of liver-related events compared to nonpregnant women with cirrhosis is unclear. This study evaluates the association between pregnancy and liver-related outcomes in women with compensated cirrhosis. *Approach and Results*. Population-based retrospective matched cohort study in Ontario, Canada, using routinely collected healthcare data. Pregnant women with compensated cirrhosis and without prior history of decompensation between 2000 and 2016 were identified and matched to nonpregnant women with compensated cirrhosis on age, etiology of cirrhosis, and socioeconomic status in a 1 : 2 ratio. The association between pregnancy and the composite outcome of nonmalignant decompensation, liver transplant (LT), and death up to two years after cohort entry was estimated using the multivariate Cox proportional hazard regression adjusting for potential confounders. Overall, 5,403 women with compensated cirrhosis were included (1,801 pregnant; 3,602 nonpregnant; median age 31 years (IQR 27-34); 60% nonalcoholic fatty liver disease, 34% viral hepatitis). After two years of follow-up, only 19 (1.1%) pregnant women had a liver-related event compared to 319 (8.9%) nonpregnant women. Pregnant women with compensated cirrhosis had a lower hazard of a liver-related event compared to nonpregnant women (aHR 0.14, 95% CI 0.09-0.22, *P* < .001).

**Conclusions:**

Pregnancy in women with compensated cirrhosis is not associated with increased liver-related events compared to nonpregnant women. These results can facilitate counselling women with cirrhosis of child-bearing age and suggests that pregnancy may not accelerate liver disease progression.

## 1. Introduction

The incidence and prevalence of cirrhosis have increased in North America and importantly have grown most in women of child-bearing age [1]. Several studies have documented rising numbers of pregnancies occurring in women with cirrhosis [2–4], with rates of childbirth in those with compensated disease similar to the general population of reproductive-aged women [1–3]. It is well established that adverse perinatal outcomes such as preterm delivery, induction of labor, intrahepatic cholestasis of pregnancy, and neonatal respiratory distress are more common in pregnant women with cirrhosis compared to women without cirrhosis [2–7]. However, studies evaluating the association between pregnancy and liver-related events including decompensation, need for liver transplant (LT), and death have been limited. Historically, liver-related events during pregnancy were as high as 50% [8], yet more contemporary population-level data has suggested that these events occur in less than 10% of pregnancies [2, 3]. Higher model for end-stage liver disease (MELD) scores [9], and a history of preconception hepatic decompensation has been associated with a higher risk of liver-related event among pregnant women with cirrhosis [3]. Whether pregnancy actually alters the natural history of cirrhosis compared to nonpregnant women with cirrhosis is less well established [2].

Pregnancy is associated with physiologic changes which can lead to increases in portal pressure, and in women with cirrhosis, variceal bleeding has been reported to be the most common decompensation event during pregnancy [8, 10]. Yet, pregnancy also represents a state of immune modulation [11] and increased serum levels of estrogen and progesterone, which may influence the natural history of chronic liver disease [10–12]. Indeed, women with autoimmune hepatitis have lower risk of disease flare in pregnancy as compared to the postpartum period [12], and the biochemical indices of hepatic function measured preconception have been shown to be unchanged when compared to postpartum [13]. The natural history of cirrhosis among women of reproductive age is unclear given studies describing outcomes have mainly been comprised of middle-aged men with underlying alcohol or viral associated cirrhosis. Therefore, comparing event rates among pregnant women with cirrhosis to historic data is not applicable. Additionally, no studies have described the impact of pregnancy on the natural history of cirrhosis.

The aim of this study was to describe the association between pregnancy and liver-related decompensation events, liver transplant (LT), and death in pregnant women with compensated cirrhosis compared to nonpregnant women with compensated cirrhosis.

## 2. Methods

### 2.1. Study Design and Databases

We conducted a population-based retrospective matched cohort study using data from universal healthcare coverage in the province of Ontario, Canada, from 01/01/2000 to 12/31/2018 housed at ICES (http://www.ices.on.ca/). ICES is an independent, nonprofit research institute whose legal status under Ontario's health information privacy law allows it to collect and analyze healthcare and demographic data, without informed consent, for health system evaluation and improvement. Ontario provides universal healthcare coverage for its diverse population of approximately 14 million people under the Ontario Health Insurance Plan (OHIP). The primary databases used are outlined in supplemental Table 1 and include the Registered Persons Database (RPDB) for demographic and vital status information, Canadian Institute for Health Information Discharge Abstract Database (CIHI DAD) for diagnostic and procedural information from all hospitalizations, National Ambulatory Care Reporting System (NACRS) for diagnostic and procedural information from outpatient and emergency room encounters, the OHIP Physician Claims Database which includes all claims made by physicians for universally insured services, Ontario Laboratory Information System (OLIS), and the MOMBABY dataset which links the CIHI DAD records of delivering mothers and their newborns and includes data on the date of delivery and gestational weeks at delivery for all pregnancies of at least 20-week gestation. These datasets were linked using unique encoded and analyzed at ICES. The study protocol was approved by the Queen's University Health Sciences Research Ethics Board. This study was designed, analyzed, and reported in accordance with the Strengthening the Reporting of Observational Studies in Epidemiology (STROBE) statement.

### 2.2. Study Cohort and Matching

Women with cirrhosis were identified using a validated case definition of one inpatient or outpatient OHIP or International Classification of Diseases (ICD), 9th Revision (ICD-9) or 10th Revision (ICD-10) code for cirrhosis or nonbleeding esophageal varices. This case definition has a sensitivity of 99%, specificity 77%, positive predictive value 88%, and negative predictive value 98% [14]. Women with a history of decompensation prior to or at cohort entry were excluded because the study outcome was liver-related events including decompensation. These exclusion events were identified using a validated case definition as a composite of hepatic encephalopathy, ascites, variceal hemorrhage, hepatorenal syndrome, and liver failure with an associated sensitivity and specificity of 89% each [14]. Individuals were also excluded if they lacked a unique identifier, <1 year OHIP eligibility before delivery, or had a history of liver transplantation (LT).

Women with cirrhosis and a pregnancy carried to at least 20-week gestation occurring after cirrhosis diagnosis were identified by linkage to the MOMBABY dataset. In women who had >1 pregnancy during the study period, the first pregnancy was included in the cohort and follow-up time was censored at the date of conception of the subsequent pregnancy. Pregnant women with cirrhosis were matched at the time of conception to nonpregnant women at cirrhosis index in a 1 : 2 ratio on age (±5 years), cirrhosis etiology, and socioeconomic status (SES) [15]. Age at conception (pregnant women) or cirrhosis diagnosis (nonpregnant women) was obtained from the RPDB. Both groups were followed for up to 2 years from index date, with index date being time of conception for pregnant women with cirrhosis and date of cirrhosis diagnosis for nonpregnant women.

### 2.3. Demographics, Covariates, and Outcomes

Cirrhosis etiology was determined using a validated hierarchical algorithm to categorize individuals as either nonalcoholic fatty liver disease (NAFLD), viral hepatitis (both hepatitis B and C), alcohol-associated liver disease (ALD), or autoimmune liver disease (AI)/other as previously described [16]. Socioeconomic status (SES) was defined using postal codes in the RPDB and described as income quintiles from Statistics Canada. Underlying comorbid illness was described using the Charlson comorbidity index (CCI) using a one-year look-back window [17]. A history of obesity (OHIP code and ICD-9 278; ICD-10 E66) or infertility (OHIP code 628) within five years of cohort entry was described. Parity was determined by childbirth events in the MOMBABY dataset prior to cohort entry. Overall physician visits were determined one year prior to and up to two years after cohort entry by OHIP billing codes. Physician visits by either a gastroenterologist or internal medicine physician were identified. Where available, prepregnancy model for end-stage liver disease (MELD) score [18] and platelet count within one year of cohort entry were described using OLIS. Diagnostic upper endoscopy after cirrhosis diagnosis was identified with OHIP codes Z399 and Z515. The primary outcome was liver-related events defined as any one of the following: hepatic decompensation as defined above, LT identified using OHIP billing codes, or death identified by the date of death from the RPDB. Women were censored once an outcome event occurred. In the situation where two or more outcome events occurred during the follow-up period (i.e., decompensation then LT), women were censored after their first outcome event.

### 2.4. Statistical Approach

The baseline demographics of the cohort were described with medians and proportions as appropriate and compared by pregnancy status. The Cox proportional hazard regression models were used to assess the association between pregnancy and liver-related events, adjusting for comorbid illness with the CCI and history of obesity. The model was also adjusted for history of infertility (yes vs. no) and previous parity (yes vs. no) as instrumental variables as potential indicators of active participation in strategies to manage chronic liver disease [19]. The proportional hazard assumption was confirmed by visual inspection of the Kaplan-Meier curves over the study period. The robust variance estimator was used to account for the matched design. All analyses were performed by using SAS, version 9.4 (SAS Institute, Cary, NC). Because of an ICES privacy agreement, data containing small cells (*n* < 6) are not reportable due to reidentification risk.

## 3. Results

**Figure 1:**
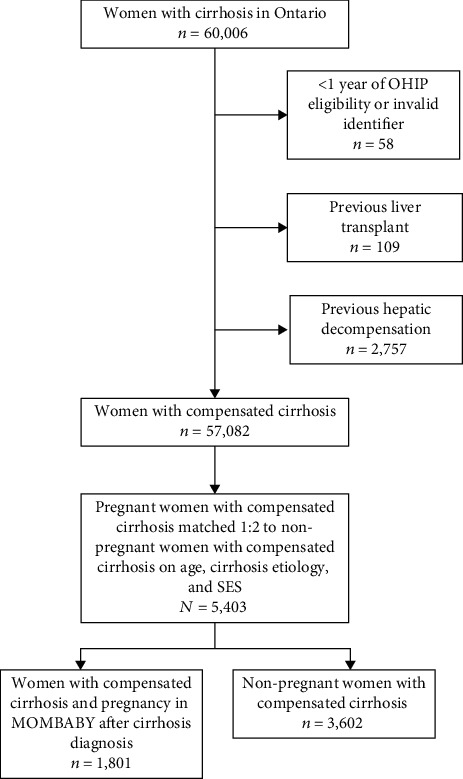
Inclusion and exclusion criteria for creation of cohort of pregnant women with cirrhosis matched 1 : 2 to nonpregnant women with cirrhosis.

The baseline demographics of the cohort are outlined in Table 1. A total of 5,403 women with compensated cirrhosis were included in this study; 1,801 were pregnant and 3,602 were not pregnant (Figure 1). The median age at cohort entry was 31 years (IQR 27-35). The majority of women had NAFLD as the underlying etiology of cirrhosis (60%), followed by viral hepatitis (35%) and ALD (4%). Overall, there was a higher proportion of women in the lower SES quintiles compared to higher income quintiles. There was a higher proportion of women with comorbid illness as identified with a CCI > 0 in the nonpregnant group (7%) compared to the pregnant group (4%). A higher proportion of women with a history of infertility and parity were observed in those who were pregnant compared to those who were not. In patients with available data (20.6%), MELD scores were slightly lower in pregnant women compared to nonpregnant women (median MELD 6 vs. 7); however, platelet counts were similar (median 245 vs. 250). Most women had at least one physician visit both one year prior to and during the study period; however, there was a higher proportion of women who had visits with either gastroenterology or internal medicine in nonpregnant women compared to pregnant women. Diagnostic upper endoscopy during the study period was more frequent in women who were not pregnant (30% vs. 24%).

A total of 19 (1.1%) pregnant women had a liver-related event during the 2-year study period compared to 319 (8.9%) in nonpregnant women. The most common liver-related event in pregnancy was variceal hemorrhage (*n* = 13, 68%); per ICES privacy policy, small cell numbers prevented detailing other adverse outcomes. In nonpregnant women, the most common events were ascites (*n* = 98, 30.7%), hepatorenal syndrome/hepatic failure (*n* = 73, 22.8%), and variceal hemorrhage (*n* = 61, 19.1%). In pregnant women, <6 died or received LT during follow-up and none occurred prior to childbirth, while in the nonpregnant group, 56 women (1.6%) died and 19 (0.5%) received LT.

A Cox proportional hazard regression model was used to evaluate the association between pregnancy and liver-related events with adjustment for CCI, obesity, parity, and infertility (Table 2). The results from the multivariate model suggests that in women with compensated cirrhosis, pregnancy is associated with an 86% reduced hazard for decompensation, LT, or death (aHR: 0.14, 95% CI: 0.09-0.22, *P* < .001).

## 4. Discussion

In this large population-based cohort study of women with compensated cirrhosis, pregnancy was associated with a substantially lower hazard of a liver-related event compared to age, SES, and etiology matched women with compensated cirrhosis who were not pregnant. These results suggest that pregnancy is not associated with a higher risk of liver-related events, in women with compensated cirrhosis. Taken together, these results provide important information for both women with cirrhosis who are contemplating pregnancy and for providers caring for women with cirrhosis who wish to become or who are pregnant.

Limited data exist on the impact of pregnancy on the natural history of cirrhosis. Our results are in keeping with a recent study from the United Kingdom demonstrating that in a cohort of pregnant women with chronic liver disease, the MELD score, MELD-Na, AST-to-platelet ratio index, and albumin-bilirubin index measured both preconception and postpartum were similar [13]. Although rates of decompensation among reproductive aged women with cirrhosis prior to this study were unknown, our observed decompensation event rate in the nonpregnant women with cirrhosis of 7% over two years is similar to expected annual rates of decompensation in historic cohorts with compensated cirrhosis [20, 21]. The lower rate of decompensation in pregnant women may be related by several different factors. Firstly, liver function is likely better preserved in women with cirrhosis who are able to achieve pregnancy. Confounding due to severity of liver disease is a concern despite attempts to adjust for such confounding by matching for age and cirrhosis etiology and adjusting for the CCI. A higher proportion of nonpregnant women were engaged in care with either gastroenterology or internal medicine both prior to and during the study period which may also reflect more advanced liver disease. Nonpregnant women were also more likely to have a diagnostic upper endoscopy than pregnant women potentially reflecting clinical evidence of underlying portal hypertension. Among the 20% of patients with available lab data for calculation of MELD score, the median value was statistically lower in pregnant (6; IQR 6-7) versus nonpregnant women (7; IQR 6-9) though, likely not clinically different. Secondly, pregnant women may have been more likely to engage in efforts to control their underlying liver disease such as antiviral therapy, alcohol abstinence, and lifestyle changes such as weight loss and healthy eating, which all could impact liver-related events. Unfortunately, these details are not available in the administrative data and could not be accounted for. However, we observed that pregnant women with cirrhosis were more likely to have a history of previous childbirth and infertility suggesting that this group of women may have been actively pursuing pregnancy and in turn more likely to participate in the interventions mentioned. Thus, while our results are reassuring for patients and their providers, additional prospective studies that can assess disease severity and interventions during pregnancy would be beneficial.

It is well known that the physiologic changes associated with pregnancy including increased blood volume and pressure in the abdominal cavity during gestation in women with cirrhosis can result in worsening portal hypertension [22]. A recent meta-analysis has suggested that variceal bleeding is the most common liver event among pregnant women with cirrhosis occurring in ~4% when both women with and without decompensated disease are included [7]. Indeed, in our cohort of women with only compensated disease, the most common liver-related event in those who were pregnant was variceal hemorrhage which occurred in ~1% and overall was responsible for 64% of liver events, considerably more frequent compared to women who were not pregnant (19%). However, the overall proportion of pregnant women with compensated cirrhosis who had a liver-related event (1%) within the two-year study period was much lower than would be expected. Therefore, the question remains whether the pregnant state itself could be playing a role in preserving liver synthetic function. Data on fetal microchimerism has shown fetal progenitor cells persisting in maternal circulation and tissue during pregnancy and postpartum that may proliferate in the liver [23], and these cells have been shown to migrate to areas of damaged hepatocytes in patients with hepatitis C and autoimmune thyroid disease [24, 25]. The possibility that this may influence hepatic decompensation is intriguing. Other physiologic changes in pregnancy including increases in female sex hormones [26] and immune “modulated” state associated with pregnancy may also contribute. There have been several published studies which have evaluated the potential impact of estrogen and its protective nature in liver disease. One study in individuals with NAFLD observed that men and postmenopausal women were at increased risk of having more severe liver fibrosis and that estrogen replacement in postmenopausal women may decrease the risk of advanced fibrosis [27]. Additionally, a study evaluating pregnancies in women with autoimmune hepatitis found that women were less likely to have disease flares during pregnancy compared to the postpartum period which is hypothesized to be related to the altered immune milieu during pregnancy [12]. Taken together, pregnancy may be protective in chronic liver disease, and this, at least partially, also helps to explain the lower frequency of liver-related events we observed in our population.

The strengths of our study include the inclusion of a large contemporary cohort of young women with compensated cirrhosis of varying etiologies and SES, with reliable and validated outcome ascertainment given the use of routinely collected population-level healthcare data. However, several limitations must be acknowledged. First are biases inherent to the retrospective and administrative nature of the study design including misclassification and underreporting but are unlikely to be differential based on pregnancy status. Secondly, only pregnancies that were carried beyond 20 weeks were evaluated as early fetal loss is not recorded in the MOMBABY dataset. Therefore, we are unable to describe the impact of pregnancy on liver-related outcomes occurring before 20-week gestation. However, this is unlikely to significantly impact the rates of decompensation, as most physiologic changes that influence portal hypertension occur in the second trimester with most pregnancy associated liver complications previously reported to occur beyond the second trimester [26]. Third, our approach to match on age resulted in pregnant women having diagnosed cirrhosis for longer than nonpregnant women. Potential bias from our matching approach would favor more adverse study outcomes in those who were pregnant given that they had diagnosed disease for longer than those who were not pregnant. In an ideal setting, we would have also been able to match women based on MELD score at index to ensure similar hepatic function. However, given the nature of the data used, this was not possible due to incomplete data in OLIS. Therefore, it remains possible that despite only including women without a history of decompensation, those who were not pregnant may have had more advanced disease than those who were pregnant. Finally, the ICES data do not contain information on an individual's race or medication usage, and therefore, these could not be described or accounted for in this study. Although Ontario has a racially diverse population with ~25% self-identifying as a visible minority, its external validity needs to be considered when applying these findings to other populations.

In conclusion, pregnancy in women with compensated cirrhosis is not associated with increased risks of liver-related events compared to nonpregnant women. Although confounding from better health status in women who were able to conceive may have affected our observed differences, these results should nonetheless help counselling of women and provides important contemporary information for multidisciplinary teams involved in the care of pregnant women with cirrhosis to guide preconception counselling and clinical guidelines for this population.

## Figures and Tables

**Table 1:** Baseline demographics of the matched cohort of women with cirrhosis stratified by pregnancy status.

	Overall *n* = 5,403	Nonpregnant *n* = 3,602	Pregnant *n* = 1,801	^*P* value
Age, median years (IQR)	31 (27-34)	31 (27-34)	31 (27-34)	.722
Cirrhosis etiology, *n* (%)				>.999
NAFLD/cryptogenic	3,243 (60.0)	2,162 (60.0)	1,081 (60.0)	
Viral hepatitis	1,887 (34.9)	1,258 (34.9)	629 (34.9)	
ALD	189 (3.5)	126 (3.5)	63 (3.5)	
Other^∗^	84 (1.6)	56 (1.6)	28 (1.6)	
Income quintile, *n* (%)				.861
1 (lowest)	1,302 (24.1)	871 (24.2)	431 (23.9)	
2	1,054 (19.5)	708 (19.7)	346 (19.2)	
3	1,187 (22.0)	793 (22.0)	394 (21.9)	
4	1,077 (19.9)	712 (19.8)	365 (20.3)	
5 (highest)	738 (13.7)	492 (13.7)	246 (13.7)	
Missing	45 (0.8)	26 (0.7)	19 (1.1)	
CCI, *n* (%)				<.001
0	5,083 (94.1)	3,351 (93.0)	1,732 (96.2)	
1	186 (3.4)	133 (3.7)	53 (2.9)	
2	54 (1.0)	48 (1.3)	6 (0.3)	
3+	80 (1.5)	70 (1.9)	10 (0.6)	
Obesity, *n* (%)	599 (11.1)	420 (11.7)	179 (9.9)	.057
History of infertility	649 (12.0)	253 (7.0)	396 (22.0)	<.001
Parity				<.001
Nulliparous	3,403 (63.0)	2,517 (69.9)	886 (49.2)	
Parous	2,000 (37.0)	1,085 (30.1)	915 (50.8)	
MELD score, median (IQR)	6 (6-8)	7 (6-9)	6 (6-7)	<.001
Available, *n* (%)	1,115 (20.6)	804 (22.3)	311 (17.3)	
Platelet count (E^9^/L), median (IQR)	248 (205-297)	250 (203-301)	245 (207-291)	0.291
Available, *n* (%)	3,033 (56.1)	1,885 (52.3)	1,148 (63.7)	
EGD after cirrhosis diagnosis, *n* (%)	1,509 (27.9)	1,086 (30.1)	423 (23.5)	<.001
Healthcare visit 1 year prior, *n* (%)				<.001
Any physician	5,363 (99.3)	3,602 (100)	1,761 (97.8)	
Gastroenterology	2,319 (42.9)	1,780 (49.4)	539 (29.9)	
Internal medicine	1,394 (25.8)	1,099 (30.5)	295 (16.4)	
Healthcare visit during study, *n* (%)				
Any physician	5,394 (99.8)	3,593 (99.8)	1,801 (100)	.034
Gastroenterology	2,240 (41.5)	1,813 (50.3)	427 (23.7)	<.001
Internal medicine	1,648 (30.5)	1,260 (35.0)	388 (21.5)	<.001
Liver-related events, *n* (%)				<.001
Overall	338 (6.3)	319 (8.9)	19 (1.1)	
Decompensation	^∗∗^	244 (6.8)	^∗∗^	
Liver transplant	^∗∗^	19 (0.5)	^∗∗^	
Death	^∗∗^	56 (1.6)	^∗∗^	

^∗^Includes autoimmune hepatitis, Wilson's disease, hereditary hemochromatosis, primary sclerosing cholangitis, primary biliary cholangitis, and alpha-1 antitrypsin deficiency; ^*P* values compare pregnant to nonpregnant women; ^∗∗^Cells suppressed due to risk of reidentification. NAFLD: nonalcoholic fatty liver disease; ALD: alcohol-related disease; CCI: Charlson comorbidity index; IQR interquartile range; MELD: model for end-stage liver disease; EGD: upper endoscopy.

**Table 2:** Cox proportional hazard regression analysis for the outcome of a liver-related event during follow-up.

	Unadjusted			Adjusted		
	HR	95% CI	*P* value	HR	95% CI	*P* value
Pregnant (vs. not pregnant)	0.11	0.07-0.18	<.001	0.14	0.09-0.22	<.001
CCI (per 1 point increase)	1.63	1.53-1.74	<.001	1.53	1.43-1.64	<.001
Obese (yes vs. no)	0.26	0.14-0.48	<.001	0.27	0.15-0.50	<.001
Infertility (yes vs. no)	0.54	0.42-0.69	<.001	0.71	0.56-0.92	.009
Previous parity (yes vs. no)	0.43	0.27-0.68	<.001	0.65	0.41-1.02	.062

HR: hazard ratio; CCI: Charlson comorbidity index.

## Data Availability

The dataset from this study is held securely in coded form at ICES. While legal data sharing agreements between ICES and data providers (e.g., healthcare organizations and government) prohibit ICES from making the dataset publicly available, access may be granted to those who meet prespecified criteria for confidential access, available at http://www.ices.on.ca/DAS (email: das@ices.on.ca). The full dataset creation plan and underlying analytic code are available from the authors upon request, understanding that the computer programs may rely upon coding templates or macros that are unique to ICES and are therefore either inaccessible or may require modification.

## Abbreviations

LT: Liver transplant
MELD: Model for end-stage liver disease
NAFLD: Nonalcoholic fatty liver disease
OHIP: Ontario Health Insurance Plan
RPDB: Registered Persons Database
CIHI DAD: Canadian Institute for Health Information Discharge Abstract Database
NACRS: National Ambulatory Care Reporting System
OLIS: Ontario Laboratory Information System
STROBE: Strengthening the Reporting of Observational Studies in Epidemiology
ICD: International Classification of Diseases
SES: Socioeconomic status
ALD: Alcohol-related liver disease
AI: Autoimmune liver disease
CCI: Charlson comorbidity index
HCC: Hepatocellular carcinoma.

## Additional Points

*Lay Summary*. In this large population-based study of women with compensated cirrhosis of child-bearing age, pregnancy did not increase the risk of having a liver-related event. This information will help to guide evidence-based discussions regarding family planning in women with cirrhosis.

## Supplementary Materials

Supplementary Materials Supplemental Table 1: ICES databases used for the study.
