# Supplementary material for: Pregnancy Is Not Associated with an Increased Risk of Decompensation, Transplant, or Death in Compensated Cirrhosis
Source: Int J Hepatol. 2022 Jul 6;2022:9985226. doi: 10.1155/2022/9985226 (PMC9279084; doi:10.1155/2022/9985226)
Supplement: Supplementary Materials — Supplemental Table 1: ICES databases used for the study. [file 9985226.f1.docx]

| **Supplemental Table 1.** ICES Databases Used for the Study | | |
| --- | --- | --- |
| **Database** | **Contents** | **Use in the study variables** |
| Registered Persons Database  (RPDB) | Demographic and vital status information for individuals covered under OHIP | Date of birth, date of death |
| Canadian Institute for Health Information Discharge  (CIHI-DAD) | Captures diagnostic and procedural information from inpatient hospital admissions | Cirrhosis; cause of cirrhosis;  liver decompensation  and liver-related  outcomes; CCI |
| National Ambulatory Care Reporting System  (NACRS) | Captures diagnostic and procedural information from ambulatory care and emergency room visits | Cirrhosis, cause of cirrhosis,  liver decompensation |
| OHIP Physician Claims Database | Claims made by physicians for universally insured services | Cirrhosis, cause of cirrhosis and liver decompensation |
| Ontario Laboratory Information System (OLIS) | Contains data on laboratory testing from a proportion of inpatient and outpatient laboratory tests in Ontario from 2007-2015 | MELD score and platelet counts |
| MOMBABY | Contains information including perinatal outcomes from routine mother-infant linkage if infants were carried to at least 20 weeks’ gestation | Cohort identification |
| CCI: Charlson co-morbidity index | | |
